# Correlation of Pandemic (H1N1) 2009 Viral Load with Disease Severity and Prolonged Viral Shedding in Children

**DOI:** 10.3201/eid1608.091918

**Published:** 2010-08

**Authors:** Chung-Chen Li, Lin Wang, Hock-Liew Eng, Huey-Ling You, Ling-Sai Chang, Kuo-Shu Tang, Ying-Jui Lin, Hsuan-Chang Kuo, Ing-Kit Lee, Jien-Wei Liu, Eng-Yen Huang, Kuender D. Yang

**Affiliations:** Author affiliation: Chang Gung Memorial Hospital–Kaohsiung Medical Center, Kaohsiung, Taiwan; and Chang Gung University College of Medicine, Kaohsiung

**Keywords:** Pandemic (H1N1) 2009 virus, viruses, respiratory infections, influenza, viral load, viral shedding, pneumonia, oseltamivir treatment, research

## Abstract

Younger children may require a longer isolation period and more aggressive treatment.

## MEDSCAPE CME

Medscape, LLC is pleased to provide online continuing medical education (CME) for this journal article, allowing clinicians the opportunity to earn CME credit. This activity has been planned and implemented in accordance with the Essential Areas and policies of the Accreditation Council for Continuing Medical Education through the joint sponsorship of Medscape, LLC and Emerging Infectious Diseases. Medscape, LLC is accredited by the ACCME to provide continuing medical education for physicians. Medscape, LLC designates this educational activity for a maximum of 0.5 *AMA PRA Category 1 Credits*™. Physicians should only claim credit commensurate with the extent of their participation in the activity. All other clinicians completing this activity will be issued a certificate of participation. To participate in this journal CME activity: (1) review the learning objectives and author disclosures; (2) study the education content; (3) take the post-test and/or complete the evaluation at http://cme.medscape.com/viewpublication/30063; (4) view/print certificate.

## Learning Objectives

- Identify the most common clinical manifestations associated with pandemic (H1N1) 2009 infection.
- Recognize different diagnostic tests for pandemic (H1N1) 2009 infection.
- Identify independent predictors of viral shedding in pandemic (H1N1) 2009 infection, including impact of age and comorbidities.

## Editor

**Carol Snarey, MA**, Copyeditor, Emerging Infectious Diseases. Disclosure: Carol Snarey, MA, has disclosed no relevant financial relationships.

## CME AUTHOR

**Desiree Lie, MD, MSED**, Clinical Professor of Family Medicine, Director of Research and Faculty Development, University of California, Irvine at Orange, California. *Disclosure: Désirée Lie, MD, MSEd, has disclosed the following relevant financial relationship: served as a nonproduct speaker for "Topics in Health" for Merck Speaker Services.*

## AUTHORS

Disclosures: Chung-Chen Li, MD; Lin Wang, MD; Hock-Liew Eng, MD; Huey-Ling You, MD; Ling-Sai Chang, MD; Kuo-Shu Tang, MD; Ying-Jui Lin, MD; Hsuan-Chang Kuo, MD; Ing-Kit Lee, MD; Jien-Wei Liu, MD; Eng-Yen Huang, MD; and Kuender D. Yang, MD, have disclosed no relevant financial relationships.
