# Supplementary material for: Correlation of Pandemic (H1N1) 2009 Viral Load with Disease Severity and Prolonged Viral Shedding in Children
Source: Emerg Infect Dis. 2010 Aug;16(8):1265–72. doi: 10.3201/eid1608.091918 (PMC3298297; doi:10.3201/eid1608.091918)
Supplement: Related texts

# Research article: Correlation of Pandemic (H1N1) 2009 Viral Load with Disease Severity and Prolonged Viral Shedding in Children

**DOI:** 10.3201/eid1608.091918

**Authors:** Chung-Chen Li, Lin Wang, Hock-Liew Eng, Huey-Ling You, Ling-Sai Chang, Kuo-Shu Tang, Ying-Jui Lin, Hsuan-Chang Kuo, Ing-Kit Lee, Jien-Wei Liu, Eng-Yen Huang, Kuender D. Yang

**Affiliations:** Author affiliation: Chang Gung Memorial Hospital–Kaohsiung Medical Center, Kaohsiung, Taiwan; and Chang Gung University College of Medicine, Kaohsiung

## Abstract

Pandemic (H1N1) 2009 virus causes severe illness, including pneumonia, which leads to hospitalization and even death. To characterize the kinetic changes in viral load and identify factors of influence, we analyzed variables that could potentially influence the viral shedding time in a hospital-based cohort of 1,052 patients. Viral load was inversely correlated with number of days after the onset of fever and was maintained at a high level over the first 3 days. Patients with pneumonia had higher viral loads than those with bronchitis or upper respiratory tract infection. Median viral shedding time after the onset of symptoms was 9 days. Patients <13 years of age had a longer median viral shedding time than those >13 years of age (11 days vs. 7 days). These results suggest that younger children may require a longer isolation period and that patients with pneumonia may require treatment that is more aggressive than standard therapy for pandemic (H1N1) 2009 virus.

The influenza A pandemic (H1N1) 2009 virus, initially identified in Mexico and the United States in April 2009, has been reported in >213 countries (*1*). Severe illness and death can occur in humans infected with this virus, particularly young persons and pregnant women (*2**–**5*). Recent data from the United States showed that almost half of hospitalized case-patients were children <18 years of age and suggested that antiviral drugs were beneficial in these patients, especially when initiated early (*6*). This finding implies that the successful control of viral replication by using antiviral drugs is associated with a good clinical outcome.

Because viral replication is necessary for disease pathogenesis in other influenza infections (*7**,**8*), information on the correlation between viral load and the clinical spectrum of illnesses among persons infected with pandemic (H1N1) 2009 virus is emerging. However, viral replication patterns and the effect of antiviral drugs on viral load have not been adequately studied.

We undertook the present study to characterize the kinetic changes in viral load and shedding in a hospital-based cohort by real-time reverse transcription–PCR (RT-PCR) and to analyze the factors that influence the rate of viral RNA clearance. A correlation between the virologic profile and the clinical features of pandemic (H1N1) 2009 virus–infected patients would provide essential information for epidemiologic control and clinical management in terms of antiviral therapy and infection control approaches.

## Materials and Methods

### Study Design and Study Subjects

The outbreak of pandemic (H1N1) 2009 in Taiwan began in July 2009. Our hospital, a 2,600-bed medical center that had experienced an outbreak of severe acute respiratory syndrome in 2003 (*9**–**12*), reorganized the severe acute respiratory syndrome research team to study pandemic (H1N1) 2009 virus in Taiwan. We established fever clinics (also called flu-like illness clinics) after a seriously ill child died of a pandemic (H1N1) 2009 virus infection at the beginning of this outbreak. Throat (tonsillopharyngeal) swab specimens from patients with influenza-like symptoms were obtained for the diagnosis of pandemic (H1N1) 2009 virus infection by differential and quantitative reverse RT-PCR or virus culture and RT-PCR. Patients confirmed as infected with pandemic (H1N1) 2009 virus received oseltamivir treatment for 5 days in a dosage based on body weight, according to the manufacturer’s recommendations.

Respiratory specimens were serially collected during the period of hospitalization or upon outpatient follow-up after informed consent was obtained. We collected the following clinical data: demographic characteristics, disease severity, coexisting conditions, fever-onset time, time to initiate antiviral treatment, and duration of fever after antiviral treatment. For the classification of disease severity, pneumonia was defined by the presence of patchy alveolar opacities on chest radiographs, and meningoencephalitis was diagnosed on the basis of presence of brain swelling with leptomeningeal enhancement seen on magnetic resonance imaging. This study was approved by the Institutional Review Board of Chang Gung Memorial Hospital.

### Virus Culture

Virus culture was performed by using a Madin-Darby canine kidney cell line obtained from American Type Culture Collection (Manassas, VA, USA). Supernatants from infected cultures were mixed with equal volumes of 1% guinea pig erythrocyte suspension and incubated for 1 h at 4°C. Culture cells with supernatants found positive by hemagglutination test were subjected to a direct fluorescent antibody assay with fluorescein-conjugated monoclonal antibodies against influenza viruses A and B and Evans blue dye (Oxoid, Ely, UK) to differentiate between influenza A and B viruses. Influenza A viruses were further differentiated from novel or seasonal influenza A viruses by real-time RT-PCR, as described below.

### Differentiation and Quantification of Pandemic (H1N1) 2009 Virus

We extracted total nucleic acids from throat swab specimens or the supernatant of positive virus cultures with the Roche MagNA Pure Compact System (Roche Molecular Diagnostics, Mannheim, Germany) by using the manufacturer’s external lysis protocol and extraction reagents (Total Nucleic Acid Isolation Kit; Roche Molecular Diagnostics) to yield 100 μL of elutes. All PCRs were performed by using the standard real-time RT-PCR procedure of the Centers for Disease Control and Prevention for swine (H1N1) influenza (*13*). Briefly, 20 μL of RT-PCR mixture containing 1× Universal PCR Master Mix, 1× MultiScribe and RNase Inhibitor, 100 nmol/L TaqMan probe, and 5 μL extracted RNA, or water for no template controls, was subjected to RT-PCR in the presence of 250 nmol/L swine influenza nucleoprotein gene-specific primers or internal control (RNase P) primers. The reactions were performed and analyzed in a ABI PRISM 7000 sequence Detection System (Applied Biosystems, Branchburg, NJ, USA) under the following conditions: 30 min at 48ºC and 10 min at 95ºC, followed by 40 cycles of 30 s at 95ºC and 1 min at 60ºC.

For the quantitative assay, a reference standard was prepared by using the pUC57 vector (GeneDireX, Las Vegas, NV, USA) containing the corresponding specific viral sequence. Tenfold dilutions equivalent to 1 to 1.0 × 10^7^ copies per reaction were prepared to generate calibration curves and to be run in parallel with the test samples. The limit of detection of this real-time RT-PCR was 1 copy/mL. The viral load in each sample was calculated and corrected by using the threshold cycle value of the internal control (RNase P) (*14*).

### Statistical Analysis

The correlation between the viral load and the number of days after the onset of symptoms was analyzed by using the Spearman rho correlation. Comparisons of the viral load among groups with different disease severity were analyzed by using Tukey post hoc multiple comparison after analysis of variance. The duration of viral shedding was calculated by using the Kaplan-Meier method and tested by the log-rank test. For multivariate analysis of the duration of viral shedding, we used the Cox proportional hazards model. All variables such as age, gender, disease severity, coexisting conditions, viral load from the first throat swab specimen, and time to initiate antiviral treatment were treated as categorized data in the multivariate analysis. The median value was taken as the cutoff value between the different groups in continuous variables such as age and viral load in the days after the onset of symptoms.

## Results

### Demographic Data for the Patients Infected with Pandemic (H1N1) 2009

From August 1 through September 30, 2009, a total of 1,052 patients were analyzed for novel influenza (H1N1) infection by RT-PCR, and pandemic (H1N1) 2009 virus infections were confirmed in 602 patients. In the 425 samples analyzed by virus culture and RT-PCR, 222 were RT-PCR positive and 204 were culture positive. Of the 204 culture-positive samples, 192 (94%) were RT-PCR positive; of the 222 RT-PCR–positive samples, 194 (87%) were culture positive. The RT-PCR method is a quantitative assay and was quicker and more sensitive than virus culture.

After 21 patients who had received oseltamivir therapy before RT-PCR diagnosis were excluded, 581 patients were monitored for involvement of other organs beyond the upper respiratory tract. Of the 581 patients, the median age was 10.1 years (range 0.38–78.8 years) (Table 1). Twenty (3.4%) patients had a severe illness, including pneumonia and meningoencephalitis. Most patients (502, 86.4%) visited the hospital for diagnosis of pandemic (H1N1) 2009 virus infection within 2 days of the onset of fever. The viral load from the first throat swab specimen was >4 log_10_ copies/mL in 278 (47.8%) patients. No deaths were found in this cohort study, but 1 patient died of severe pneumonia and cerebral hemorrhage before the start of the study.

**Table 1:** Demographic data for 581 patients with RT-PCR–confirmed pandemic (H1N1) 2009 virus infection, Taiwan*

Demographic variable	No. (%) patients†
Sex, M/F	337/244
Age, y	
Median	10.1
Range	0.38–78.8
<4	49 (8.4)
>4 to <7	87 (15.0)
>7 to <13	256 (44.1)
>13 to <18	130 (22.4)
>18 to <50	54 (9.3)
>50	5 (0.9)
Days after onset of fever	
0 (no fever)	8 (1.4)
1	354 (60.9)
2	148 (25.5)
3	49 (8.4)
4	12 (2.1)
5	7 (1.2)
>6	3 (0.5)
Disease severity	
URI†	518 (89.2)
Bronchitis	43 (7.4)
Pneumonia	17 (2.9)
Meningoencephalitis	3 (0.5)
Virus load from first throat swab, log_10_ copies/mL	
<1.0	6 (1.0)
1.0– 2.0	47 (8.1)
>2.0 –3.0	80 (13.8)
>3.0 –4.0	170 (29.3)
>4.0–5.0	190 (32.7)
>5.0– 6.0	83 (14.3)
>6.0	5 (0.9)

*RT-PCR, reverse transcription–PCR; URI, upper respiratory tract infection. †Except as indicated.

### Viral Load and Days after the Onset of Fever

Most pandemic (H1N1) 2009–infected patients visited the hospital to receive treatment for fever; the mean time to visit the hospital after the onset of fever was 1.57 days. Eight patients infected with pandemic (H1N1) 2009 virus sought treatment without fever. The mean viral load was significantly lower in afebrile patients than in febrile patients (2.61 vs. 3.82 log_10_ copies/mL, p = 0.004; Figure 1, panel A). The viral load was maintained at a high level during the first 3 days and was inversely correlated to the days after the onset of fever (Figure 1, panel B; Spearman correlation, R = –0.21, p<0.001).

**Figure 1:** A) Viral loads in afebrile and febrile patients infected with pandemic (H1N1) 2009 virus, Taiwan, before they received oseltamivir treatment. B) Correlation of the virus load with number of days after the onset of fever in febrile patients. Median, quartiles, and range are shown. Circles indicate individual values.

### Higher Viral Loads in Patients with Pneumonia

We analyzed whether the viral load was correlated with age or disease severity. The viral load in patients of all ages was not significantly different (Figure 2, panel A). We found that patients with pneumonia had significantly higher mean viral loads (4.92 log_10_ copies/mL; 95% confidence interval [CI] 4.30–5.54; n = 17) than in those with upper respiratory tract infection (3.77 log_10_ copies/mL, 95% CI 3.67–3.87; n = 518, p<0.001) or with bronchitis (3.74 log_10_ copies/mL, 95% CI 3.43–4.05; n = 43, p = 0.002; Figure 2, panel B).

**Figure 2:** Viral load before treatment in relation to age (A) and disease severity (B) in patients infected with pandemic (H1N1) 2009 virus, Taiwan. Circles indicate individual values. URI, upper respiratory tract infection. Median, quartiles, and range are shown.

### Prolonged Viral Shedding Time after Oseltamivir Treatment

Sixty patients agreed to have serial measurements taken of viral shedding after oseltamivir treatment by quantitative RT-PCR analysis of the number of viral RNA copies. Specimen collection was started from the day a participant agreed to join the study and continued in 2– 3-day intervals until viral RNA was undetectable. The results showed that the median viral shedding time was 9 days (95% CI 7.37–10.63) after the onset of symptoms, and 65% of patients had detectable influenza viral RNA for >7 days after the onset of symptoms (Figure 3, panel A). One patient with severe pneumonia had prolonged viral shedding for >27 days. The mean viral load in the throat quickly decreased from 4.13 log_10_ copies/mL to 1.15 log_10_ copies/mL from days 0–2 to days 6–8, respectively, after the onset of symptoms (Figure 3, panel B).

**Figure 3:** A) Percentage of patients with positive reverse transcription–PCR (RT-PCR) results and B) viral load in throat swabs from 60 pandemic (H1N1) 2009 virus–infected patients treated with oseltamivir, according to the number of days after the onset of symptoms, Taiwan. Median, quartiles, and range are shown**.**

### Prolonged Period of Viral Shedding in Patients <13 Years of Age

We evaluated the factors that could affect the rate of viral shedding, including age, gender, coexisting conditions, disease severity, viral load from the first throat swab specimen, and time from onset of symptoms to start of treatment. By using multivariate analysis, we found that only age was associated with a prolonged period of viral shedding (Table 2). To compare age differences, 13 years was chosen as the median cutoff age between the younger and older groups. We found that patients <13 years of age (n = 31) had a median viral shedding time of 11 days (95% CI 10.19–11.81) after the onset of symptoms; patients ages ≥13 years (n = 29) had a shorter median viral shedding time of 7 days (95% CI 5.85–8.15) after the onset of symptoms (p<0.001; Figure 4). Among patients <13 years of age, 89% had detectable influenza viral RNA for >7 days after the onset of symptoms, and 36% had detectable influenza viral RNA for >14 days (Figure 4).

**Table 2:** Factors associated with duration of viral shedding in 60 patients with pandemic (H1N1) 2009 virus infection, Taiwan*

Variable	Viral RNA detected 7 d after symptom onset, %	p value (UVA)	HR (95% CI)	p value (MVA)
Age (<13 vs. >13 y)	88.7 vs. 38.1	<0.001	4.99 (2.34–10.66)	<0.001
Sex (M vs. F)	59.6 vs. 71.5	0.650	1.18 (0.57–2.44)	0.658
Disease severity (mild vs. severe)†	60.8 vs. 77.9	0.247	1.38 (0.56–3.45)	0.487
Time to initiate antiviral treatment (<48 h vs. >48 h)	60.6 vs. 81.8	0.176	2.56 (0.88–7.69)	0.085
Viral load from the first throat swab (<4.35 vs. >4.35 log_10_ copies/mL)	64.7 vs. 55.8	0.745	1.68 (0.64–4.37	0.292
Coexisting conditions (no vs. yes)	70.7 vs. 45.5	0.402	1.85 (0.78–4.39)	0.137

*UVA, univariate analysis; MVA, multivariate analysis; HR, hazard ratio; CI, confidence interval. †Severe illness included pneumonia and meningoencephalitis; mild illness included upper respiratory tract infection and bronchitis.

**Figure 4:** Kaplan-Meier estimates of patients with positive reverse transcription–PCR (RT-PCR) results for pandemic (H1N1) 2009 infection, by age group, Taiwan.

The clinical characteristics and associated coexisting conditions for patients <13 and ≥13 years of age are shown in Table 3. No significant differences were found between the 2 groups in gender, disease severity, coexisting conditions, viral load from the first throat swab, time to initiate antiviral treatment, and duration of fever after antiviral treatment. Most of the patients in each group were afebrile within 24 h after the initiation of antiviral therapy (74.2% vs. 89.7%). Of the 60 patients, 14 (23.3%) had a coexisting condition, including 8 (25.8%) <13 years of age and 6 (20.7%) ≥13 years. Among patients ≥13 years, 1 patient had 3 underlying medical conditions (diabetes, hypertension, and congestive heart failure), and another patient had 2 conditions (hypertension and cerebrovascular disease). Asthma was the most common condition in patients <13 years of age (22.6%).

**Table 3:** Characteristics of 60 patients who were infected with pandemic (H1N1) 2009 virus, by age group, Taiwan*

Characteristic	No. (%) patients		p value†
	Age <13 years, n = 31	Age >13 years, n = 29	
Sex, M/F	14/17	15/14	0.611
Disease severity			
URI/bronchitis	22 (71.0)	22 (75.9)	0.859‡
Pneumonia	9 (29.0)	5 (17.2)	
Meningoencephalitis	0	2 (6.9)	
Duration of fever after antiviral treatment, h			
<24	23 (74.2)	26 (89.7)	0.122§
24–48	5 (16.1)	2 (6.9)	
>48	3 (9.7)	1 (3.4)	
Time to initiate antiviral treatment, h			
<48	25 (80.6)	24 (82.8)	0.833
>48	6 (19.4)	5 (17.2)	
Viral load from the first throat swab, log_10_ copies/mL			
<4.35	16 (51.6)	14 (48.3)	0.796
>4.35	15 (48.4)	15 (51.7)	
Coexisting condition			
Any 1 condition	8 (25.8)	6 (20.7)	0.640
Asthma	7	0	
Ventricular septal defect	1	0	
Hypertension	0	5	
Cerebrovascular disease	0	1	
Congestive heart failure	0	1	
Diabetes	0	1	
Pregnancy	0	1	

*URI, upper respiratory tract infection. †p values were calculated by χ^2^ test. ‡The URI/bronchitis group was compared with the combined group of pneumonia and meningoencephalitis. §Fever lasting <24 h after antiviral treatment vs. fever lasting 24 h or longer after antiviral treatment.

## Discussion

Pandemic (H1N1) 2009 virus has provided an opportunity to examine the virus-host interaction of an emerging infection. The present study demonstrated that viral load was maintained at a high level during the febrile period and was inversely correlated to the days after the onset of fever. Moreover, pandemic (H1N1) 2009 virus–infected patients with pneumonia had a higher viral load than those with bronchitis or upper respiratory tract infection. The higher viral load may be a reflection of disease severity or impaired host immunity, requiring immediate attention and aggressive treatment.

As described in previous reports (*2**,**5**,**6*), infection with the pandemic (H1N1) 2009 virus caused severe illness, including pneumonia, requiring hospitalization, and even death. More severe respiratory diseases and increased mortality have been observed in young patients (*5**,**6*). These phenomena may be partly attributed to the lack of a cross-reactive antibody response to pandemic (H1N1) 2009 virus after vaccination with recent seasonal influenza vaccines (*15*). However, apart from the cross-reactive antibody response, the level of virus load is also important in predicting disease severity in influenza infections (*7**,**8**,**16*). Further investigation to correlate the virus load with the immune response in patients infected with pandemic (H1N1) 2009 virus is necessary to clarify the pathogenesis of the disease and deaths it causes.

Studies of seasonal influenza A epidemics have demonstrated that early antiviral treatment is more effective in reducing virus load when compared with no treatment (*17**,**18*). Viral clearance is also associated with resolution of symptoms (*16**,**19*). A recent study indicated that oseltamivir-treated patients showed a greater rate of virus load reduction in nasopharyngeal aspirates than did nontreated patients when the treatment was initiated <2 days after the onset of symptoms (*20*). In the present study, after oseltamivir treatment, the mean virus load detected by RT-PCR decreased quickly from 4.13 log_10_ copies/mL to 1.15 log_10_ copies/mL, thus showing the therapeutic efficacy of oseltamivir treatment. Additional controlled trials may be necessary to clarify the clinical benefit of antiviral treatments.

Prolonged viral RNA detection has been observed in the presence of coexisting conditions, and is associated with a longer duration of illness and hospitalization in elderly patients (13.5 vs. 7.0 days) (*17*). A more prolonged period of viral shedding was found in immunosuppressed patients such as those undergoing hematopoietic stem cell transplantation (*21*); moreover, antiviral therapy substantially decreased the duration of seasonal influenza viral shedding (*22**,**23*). Children shed seasonal influenza virus for a longer period than did adults (*24*). Similar to these reports, in our study we demonstrated that the duration of viral shedding time was notably longer in patients <13 years of age than in older patients. However, why children have a longer period of viral shedding is unknown. Delayed cell-mediated immunity in children responding to a novel virus may explain, in part, their longer viral shedding time.

The median duration of time to virus detection in seasonal influenza infections is typically 7–8 days after the onset of illness, but viral shedding for up to 21 days has been reported (*24*). A recent study in China reported that the median time of pandemic (H1N1) 2009 viral shedding after the onset of symptoms, according to the results of RT-PCR, was 6 days (*25*); this finding is similar to the results of a study conducted in Singapore (*26*). However, in our study, the median shedding time was 9 days after the onset of symptoms. The longer duration of viral shedding found in our study may have been due to the younger median age (10.1 years) of participants in our study compared to those in the other studies in China (23.4 years) and Singapore (26 years). Because all of our patients received oseltamivir treatment, we could not determine the actual effect of the antiviral therapy on infection caused by the novel pandemic virus. Further studies that compare patients who have received oseltamivir therapy with those who have not may be necessary to assess the effect of antiviral treatment on viral shedding and clinical outcomes.

In conclusion, the results of our study indicate that virus load was high in the febrile period and in patients with pneumonia. Children <13 years of age had a significantly longer viral shedding period than did children ≥13 years, even after oseltamivir therapy. These results suggest that younger children may require a longer isolation period and that patients with pneumonia may require more aggressive treatment for infection with pandemic (H1N1) 2009 virus.

## References

[1] World Health Organization. Pandemic (H1N1) 2009—update 91 [cited 2010 Mar 18]. http://www.who.int/csr/don/2010_03_12/en/index.html

[2] Perez-Padilla R, de la Rosa-Zamboni D, Ponce de Leon S, Hernandez M, Quiñones-Falconi F, Bautista E, ; The INER Working Group on Influenza. Pneumonia and respiratory failure from swine-origin influenza A (H1N1) in Mexico. N Engl J Med. 2009;361:680–9. 10.1056/NEJMoa090425219564631

[3] Jamieson DJ, Honein MA, Rasmussen SA, Williams JL, Swerdlow DL, Biggerstaff MS, The Novel Influenza A (H1N1) Pregnancy Working Group. H1N1 2009 influenza virus infection during pregnancy in the USA. Lancet. 2009;374:451–8.10.1016/S0140-6736(09)61304-019643469

[4] Lister P, Reynolds F, Parslow R, Chan A, Cooper M, Plunkett A, Swine-origin influenza virus H1N1, seasonal influenza virus, and critical illness in children. Lancet. 2009;374:605–7. 10.1016/S0140-6736(09)61512-919700000

[5] Chowell G, Bertozzi SM, Colchero MA, Lopez-Gatell H, Alpuche-Aranda C, Hernandez M, Severe respiratory disease concurrent with the circulation of H1N1 influenza. N Engl J Med. 2009;381:674–9. 10.1056/NEJMoa090402319564633

[6] Jain S, Kamimoto L, Bramley AM, Schmitz AM, Benoit SR, Louie J, The 2009 Pandemic Influenza A (H1N1) Virus Hospitalizations Investigation Team. Hospitalized patients with 2009 H1N1 influenza in the United States, April–June 2009. N Engl J Med. 2009;361:1935–44. 10.1056/NEJMoa090669519815859

[7] de Jong MD, Simmons CP, Thanh TT, Hien VM, Smith GJ, Chau TN, Fatal outcome of human influenza A (H5N1) is associated with high viral load and hypercytokinemia. Nat Med. 2006;12:1203–7. 10.1038/nm147716964257PMC4333202

[8] Lowen AC, Steel J, Mubareka S, Carnero E, Garcı’a-Sastre A, Palese P. Blocking interhost transmission of influenza virus by vaccination in the guinea pig model. J Virol. 2009;83:2803–18. 10.1128/JVI.02424-0819153237PMC2655561

[9] Lee CH, Chen RF, Liu JW, Yeh WT, Chang JC, Liu PM, Altered p38 mitogen-activated protein kinase expression in different leukocytes with increment of immunosuppressive mediators in patients with severe acute respiratory syndrome (SARS). J Immunol. 2004;172:7841–7.1518716810.4049/jimmunol.172.12.7841

[10] Ko SF, Lee TY, Huang CC, Cheng YF, Ng SH, Kuo YL, Severe acute respiratory syndrome: prognostic implications of chest radiographic findings in 52 patients. Radiology. 2004;233:173–81. 10.1148/radiol.232303154715273329

[11] Lee YS, Chen CH, Chao A, Chen ES, Wei ML, Chen LK, Molecular signature of clinical severity in recovering patients with severe acute respiratory syndrome coronavirus (SARS-CoV). BMC Genomics. 2005;6:132. 10.1186/1471-2164-6-13216174304PMC1262710

[12] Chen RF, Liu JW, Yeh WT, Lee CH, Liu JW, Eng HL, Role of vascular cell adhesion molecules and leukocyte apoptosis in the lymphopenia and thrombocytopenia of patients with severe acute respiratory syndrome (SARS). Microbes Infect. 2006;8:122–7. 10.1016/j.micinf.2005.06.00716182592PMC7110783

[13] World Health Organization. CDC protocol of realtime RTPCR for swine influenza A (H1N1), version 2009 [cited 2009 May 21]. http://www.who.int/csr/resources/publications/swineflu/CDCRealtimeRTPCR_SwineH1Assay-2009_20090430.pdf

[14] Bouscambert Duchamp M, Casalegno JS, Gillet Y, Frobert E, Bernard E, Escuret V, Pandemic A(H1N1) 2009 influenza virus detection by real time RT-PCR: is viral quantification useful? Clin Microbiol Infect. 2010;16:317–21. 10.1111/j.1469-0691.2010.03169.x20121827

[15] Hancock K, Veguilla V, Lu X, Zhong W, Butler EN, Sun H, Cross-reactive antibody responses to the 2009 pandemic H1N1 influenza virus. N Engl J Med. 2009;361:1945–52. 10.1056/NEJMoa090645319745214

[16] Hayden FG, Fritz R, Lobo MC, Alvord W, Strober W, Straus SE. Local and systemic cytokine responses during experimental human influenza A virus infection. Relation to symptom formation and host defense. J Clin Invest. 1998;101:643–9. 10.1172/JCI13559449698PMC508608

[17] Lee N, Chan PK, Hui DS, Rainer TH, Wong E, Choi KW, Viral loads and duration of viral shedding in adult patients hospitalized with influenza. J Infect Dis. 2009;200:492–500. 10.1086/60038319591575PMC7110250

[18] Baccam P, Beauchemin C, Macken CA, Hayden FG, Perelson AS. Kinetics of influenza A virus infection in humans. J Virol. 2006;80:7590–9. 10.1128/JVI.01623-0516840338PMC1563736

[19] Leekha S, Zitterkopf NL, Espy MJ, Smith TF, Thompson RL, Sampathkumar P. Duration of influenza A virus shedding in hospitalized patients and implications for infection control. Infect Control Hosp Epidemiol. 2007;28:1071–6. 10.1086/52010117932829

[20] Li IW, Hung IF, To KK, Chan KH, Wong SS, Chan JF, The natural viral load profile of patients with pandemic swine-origin influenza A H1N1 2009 (pH1N1) and the effect of oseltamivir treatment. Chest. Prepublished online January 8, 2010 [cited 2010 Mar 12]. http://chestjournal.chestpubs.org/content/early/2010/01/07/chest.09-307210.1378/chest.09-3072PMC709429220061398

[21] Nichols WG, Guthrie KA, Corey L, Boeckh M. Influenza infections after hematopoietic stem cell transplantation: risk factors, mortality, and the effect of antiviral therapy. Clin Infect Dis. 2004;39:1300–6. 10.1086/42500415494906

[22] Boivin G, Coulombe Z, Wat C. Quantification of the influenza virus load by real-time polymerase chain reaction in nasopharyngeal swabs of patients treated with oseltamivir. J Infect Dis. 2003;188:578–80. 10.1086/37704612898446

[23] Ward CL, Dempsey MH, Ring CJ, Kempson RE, Zhang L, Gor D, Design and performance testing of quantitative real time PCR assays for influenza A and B viral load measurement. J Clin Virol. 2004;29:179–88. 10.1016/S1386-6532(03)00122-714962787PMC7128145

[24] World Health Organization Writing Group. Nonpharmaceutical interventions for pandemic influenza, international measures. Emerg Infect Dis. 2006;12:81–7. 10.3201/eid1201.05137016494722PMC3291414

[25] Cao B, Li XW, Mao Y, Wang J, Lu HZ, Chen YS, The National Influenza A Pandemic (H1N1) 2009 Clinical Investigation Group of China. Clinical features of the initial cases of 2009 pandemic influenza A (H1N1) virus infection in China. N Engl J Med. 2009;361:2507–17. 10.1056/NEJMoa090661220007555

[26] Ling LM, Chow AL, Lye DC, Tan AS, Krishnan P, Cui L, Effects of early oseltamivir therapy on viral shedding in 2009 pandemic influenza A (H1N1) virus infection. Clin Infect Dis. 2010;50:963–9. 10.1086/65108320180701

# Research article: Correlation of Pandemic (H1N1) 2009 Viral Load with Disease Severity and Prolonged Viral Shedding in Children

**DOI:** 10.3201/eid1608.091918

**Authors:** Chung-Chen Li, Lin Wang, Hock-Liew Eng, Huey-Ling You, Ling-Sai Chang, Kuo-Shu Tang, Ying-Jui Lin, Hsuan-Chang Kuo, Ing-Kit Lee, Jien-Wei Liu, Eng-Yen Huang, Kuender D. Yang

**Affiliations:** Author affiliation: Chang Gung Memorial Hospital–Kaohsiung Medical Center, Kaohsiung, Taiwan; and Chang Gung University College of Medicine, Kaohsiung

## Earning CME Credit

To obtain credit, you should first read the journal article. After reading the article, you should be able to answer the following, related, multiple-choice questions. To complete the questions and earn continuing medical education (CME) credit, please go to http://cme.medscape.com/viewpublication/30063. Credit cannot be obtained for tests completed on paper, although you may use the worksheet below to keep a record of your answers. You must be a registered user on Medscape.com. If you are not registered on Medscape.com, please click on the New Users: Free Registration link on the left hand side of the website to register. Only one answer is correct for each question. Once you successfully answer all post-test questions you will be able to view and/or print your certificate. For questions regarding the content of this activity, contact the accredited provider, CME@medscape.net. For technical assistance, contact CME@webmd.net. American Medical Association’s Physician’s Recognition Award (AMA PRA) credits are accepted in the US as evidence of participation in CME activities. For further information on this award, please refer to http://www.ama-assn.org/ama/pub/category/2922.html. The AMA has determined that physicians not licensed in the US who participate in this CME activity are eligible for *AMA PRA Category 1 Credits*™. Through agreements that the AMA has made with agencies in some countries, AMA PRA credit is acceptable as evidence of participation in CME activities. If you are not licensed in the US and want to obtain an AMA PRA CME credit, please complete the questions online, print the certificate and present it to your national medical association.

## Article Title: Correlation of Pandemic (H1N1) 2009 Viral Load with Disease Severity and Prolonged Viral Shedding in Children

## CME Questions

**1. A 10-year-old boy is admitted to the emergency department (ED) with fever, cough, and lethargy of approximately 24 hours' duration. The ED staff recognizes that pandemic (H1N1) 2009 infection has been prevalent in the community, and the mother reports that the child's school recently sent a note home informing parents of several cases in the school. The patient's medical history is significant for asthma, but his mother reports that he has not had wheezing or coughing with this illness and she has not used his rescue medications.**

**Given this child's age and suspected diagnosis, the clinician should be particularly alert for which of the following potential complications?**

A. Renal failure

B. Pneumonia

C. Septicemia

D. Bronchitis

**2. Which of the following diagnostic tests for pandemic (H1N1) 2009 infection would be most useful to the ED in evaluating this patient?**

A. DNA polymerase chain reaction (PCR)

B. Reverse transcription–PCR (RT-PCR)

C. Viral culture

D. Viral antibody levels

**3. Diagnostic testing confirms pandemic (H1N1) 2009; comorbidities, such as pneumonia and sepsis, are ruled out. Oseltamivir therapy is begun. The patient is monitored in the ED over the next 24 hours, and is now afebrile, alert, and taking fluids. The mother reports that she has a 2-year-old child at home who appears to be well.**

**What education should be provided to this parent about the patient's contagiousness?**

A. Viral shedding is reduced by use of oseltamivir and nondetectable within 24 hours

B. Viral shedding is higher in children under the age of 13 years, regardless of viral therapy

C. Comorbid respiratory illnesses, such as asthma, are associated with prolonged viral shedding

D. The patient's infection is not severe, and thus his period of viral shedding will be significantly shorter than average

## Activity Evaluation

**Table:** 

1. The activity supported the learning objectives.				
Strongly Disagree				Strongly Agree
1	2	3	4	5
2. The material was organized clearly for learning to occur.				
Strongly Disagree				Strongly Agree
1	2	3	4	5
3. The content learned from this activity will impact my practice.				
Strongly Disagree				Strongly Agree
1	2	3	4	5
4. The activity was presented objectively and free of commercial bias.				
Strongly Disagree				Strongly Agree
1	2	3	4	5
